# Outcomes of Third-Line Trastuzumab Deruxtecan in a Patient with De Novo Stage 4 HER2-Positive Gastric Adenocarcinoma with Enteroblastic Differentiation: A Case Report

**DOI:** 10.3390/life13091851

**Published:** 2023-08-31

**Authors:** Roger Kai-Cheong Ngan

**Affiliations:** Department of Clinical Oncology, The University of Hong Kong, Hong Kong SAR, China; roger.ngan@gleneagles.hk

**Keywords:** antibody-drug conjugate, case reports, gastric cancer, immune checkpoint inhibitors, trastuzumab

## Abstract

This case report describes the treatment of a patient diagnosed with de novo stage 4 human epidermal growth factor 2 (HER2)-positive gastric adenocarcinoma with enteroblastic differentiation (GAED), a rare and aggressive form of gastric cancer characterized by a tubulopapillary growth pattern and enteroblastic cell lineage markers such as GPC3, SALL4, and alpha fetoprotein. Given the patient’s symptomatic, advanced-stage cancer, treatment objectives were focused on effectively deterring disease progression and ameliorating symptoms throughout the anticipated multiple lines of therapy. Subsequent to standard first- and second-line therapies for HER2-positive metastatic GC, third-line treatment using the antibody-drug conjugate trastuzumab deruxtecan (T-DXd) for seven cycles resulted in satisfactory tumor control and well-preserved physical performance and quality of life, with minimal hematologic and pulmonary toxicities. The patient retained acceptable physical performance to receive subsequent lines of therapies, and still showed a tumor marker response to 5L trastuzumab-based chemotherapy. As the tumor was positive for both HER2 and programmed death-ligand 1 (PD-L1) expressions, the selection and sequencing of anti-HER2 and anti-PD-L1 therapies were discussed in relation to the latest U.S. Food and Drug Administration approvals and trial results.

## 1. Introduction

The current standard of care for human epidermal growth factor 2 (HER2)-positive (+) metastatic gastric cancer (mGC) is a combination of the anti-HER2 monoclonal antibody trastuzumab with fluoropyrimidine and a platinum agent [1]. At second line (2L), the vascular endothelial growth factor receptor-2 (VEGFR-2) inhibitor ramucirumab may be used with paclitaxel, on the basis of results from the RAINBOW study for treatment-experienced advanced GC patients [2]. However, HER2 status was not considered in RAINBOW, and <10% of enrolled patients received previous anti-HER2 or anti-epidermal growth factor receptor therapy [2].

Attempts at broadening the treatment options for HER2+ mGC had largely been unsuccessful. Upfront addition of the HER2 and epidermal growth factor receptor tyrosine kinase inhibitor lapatinib to capecitabine plus oxaliplatin in the phase 3 TRIO-013/LOGiC study did not significantly improve overall survival (OS; 12.2 vs. 9.0 months, *p* = 0.35) [3]. There was a slight improvement in progression-free survival (PFS) only when switching to non-protocol therapy before disease progression was not censored (6.0 vs. 5.4 months, *p* = 0.04) [3]. Likewise, 2L treatment with the HER2-targeted antibody-drug conjugate (ADC) trastuzumab emtansine (T-DM1) in the phase 2/3 GATSBY study failed to demonstrate superiority over physician’s choice of docetaxel or paclitaxel (median OS: 7.9 vs. 8.6 months, *p* = 0.86) [4].

After trastuzumab, the ADC T-DXd was the second HER2-directed drug to become available to mGC. In January 2021, T-DXd was approved for the treatment of trastuzumab-experienced HER2+ which was locally advanced or mGC by the U.S. Food and Drug Administration (FDA), on the basis of the phase 2, open-label DESTINY-Gastric01 trial [5,6]. T-DXd consists of the topoisomerase I inhibitor deruxtecan linked at an 8:1 ratio to trastuzumab [5]. Upon cell entry and subsequent payload cleavage, the membrane-permeable deruxtecan payload induces cytotoxic effects on both the target and neighboring cells [5,7]. Such a “bystander” effect may enhance therapeutic potency on HER2+ tumors, which tend to display intratumor heterogeneity [8]. In DESTINY-Gastric01, 187 patients were randomized 2:1 to T-DXd (n = 125) or physician’s choice of irinotecan (n = 55) or paclitaxel (n = 7). Compared with the irinotecan-or-paclitaxel group, the T-DXd group demonstrated significantly higher objective response rates (ORR; 51% vs. 14%, *p* < 0.001), as well as a significantly longer median OS (12.5 vs. 8.4 months, hazard ratio = 0.59, *p* = 0.01) [5]. With the availability of this novel therapy, it would be worthwhile to present and discuss real-world experience, including treatment planning and sequencing, toxicity management, and subsequent survival.

GAED is a rare and aggressive form of GC, and has been significantly associated with twice as frequent lymphatic and venous invasions and lymph node metastases, and ~5 times more frequent liver metastases when compared with conventional gastric adenocarcinoma [9]. Among 2,273 GCs treated at Juntendo University Hospital, Japan, between 2008 and 2017, 51 (2.2%) were GAED, including three cases that were previously diagnosed as hepatoid adenocarcinoma (HAC) [10]. Only 11 were HER2+, according to immunohistochemistry (IHC) and fluorescent in situ hybridization (FISH) criteria [10]. For these 17 early and 34 advanced cases, the 3-year overall and recurrence-free survival rates after surgery were 54% and 45%, respectively [11]. GAED patients are usually treated with surgical resection and adjuvant chemotherapy, but specific systemic therapy options have not been suggested in the literature.

Morphologically, GAED tumors exhibit yolk sac tumor-like reticular or papillary structures composed of cuboidal or columnar cells, with a glycogen-rich clear cytoplasm [9]. GAED tumor cells resemble enteroblasts, expressing enteroblastic cell lineage markers GPC3, SALL4, and alpha fetoprotein (AFP), which may contribute to the aggressive phenotype of GAED [9,12]. A major differential diagnosis is hepatoid adenocarcinoma (HAC) with clear cells, which exhibits predominantly a hepatoid instead of tubulopapillary growth pattern [13]. While the 51 cases mentioned above included HAC as a GAED subtype [9], other authors argued that HAC showed diffuse and strong expressions of GPC3, SALL4 and AFP, which may be distinguished from GAED’s focal expression of only one or two oncofetal proteins, heterogeneous SALL4 expression, and an intestinal mucin phenotype [13].

A patient with de novo stage 4 HER2+ GAED with extensive metastases began his treatment in August 2020. He developed progression after first-line (1L) treatment with S-1 (a fluoropyrimidine derivative), oxaliplatin and trastuzumab, and discontinued 2L treatment with paclitaxel + ramucirumab because of severe peripheral neuropathy. Considering the prior exposure to trastuzumab and HER2+ status, T-DXd was used as third-line (3L) treatment. This report describes and discusses the outcomes of the novel T-DXd therapy when used as 3L treatment in this patient. Given the advanced and aggressive condition, T-DXd offered satisfactory tumor control for several months while preserving quality of life, with minimal hematologic and pulmonary toxicities.

## 2. Case Report

### 2.1. Baseline Characteristics

In August 2020, a 61-year-old male presented with dyspepsia, left-lower-neck swelling and mild dysphagia. The patient was a hepatitis B carrier (hepatitis B surface antigen-positive), chronic drinker and chronic smoker. An initial blood test showed elevated AFP levels of >500 ng/mL and carcinoembryonic antigen (CEA) levels of 19.8 ng/mL. Computed tomography (CT) of the abdomen showed liver and adrenal masses, as well as enlarged mediastinal, abdominal and left supraclavicular fossa (SCF) lymph nodes (LNs).

The patient had a positron emission tomography (PET)/CT scan in September 2020 which detected a 3.4 cm tumor at the anterior wall of the stomach. There were multiple LN metastases in the upper abdomen, more than 10 liver metastases that measured up to 3.5 cm in diameter, a left adrenal metastasis, and multiple left SCF and neck LN metastases. Histopathological examination of the neck LN and esophagus–gastric junction biopsies demonstrated positive staining for AFP, SALL4 and CDX2. While CDX2-positivity suggested that hepatocellular carcinoma was unlikely, AFP- and SALL4-positivity concurred with the AFP-producing subtype of GAED. IHC for HER2 expression returned a score of 2+, indicating that there was weak-to-moderate complete, basolateral or lateral membranous reactivity. The HER2 FISH test showed clusters of HER2 signals in the majority of tumor cells, at a HER2/centromere of chromosome 17 ratio of >2.0 and a HER2/nuclei ratio of >4.0. Programmed death-ligand 1 (PD-L1) expression was also positive by IHC, at a combined positive score (CPS) of 5, which included all PD-L1-staining tumor cells, lymphocytes and macrophages in the numerator and all viable tumor cells in the denominator.

### 2.2. Treatments and Outcomes

The patient received S-1, oxaliplatin and trastuzumab as his 1L therapy (Table 1). During chemotherapy, in view of the hepatitis B carrier status, the patient was given the antiviral entecavir as prophylaxis for hepatitis B reactivation. Radiological partial response was achieved (Supplementary Figure S1). Upon clinical progression (worsening retching and heartburn, with increasing AFP and CEA levels), the patient was switched to 2L ramucirumab and paclitaxel. However, this 2L treatment was discontinued at the patient’s request because of the development of severe peripheral neuropathy that hindered ambulation and fine motor functions (Table 2).

**Figure 1:**
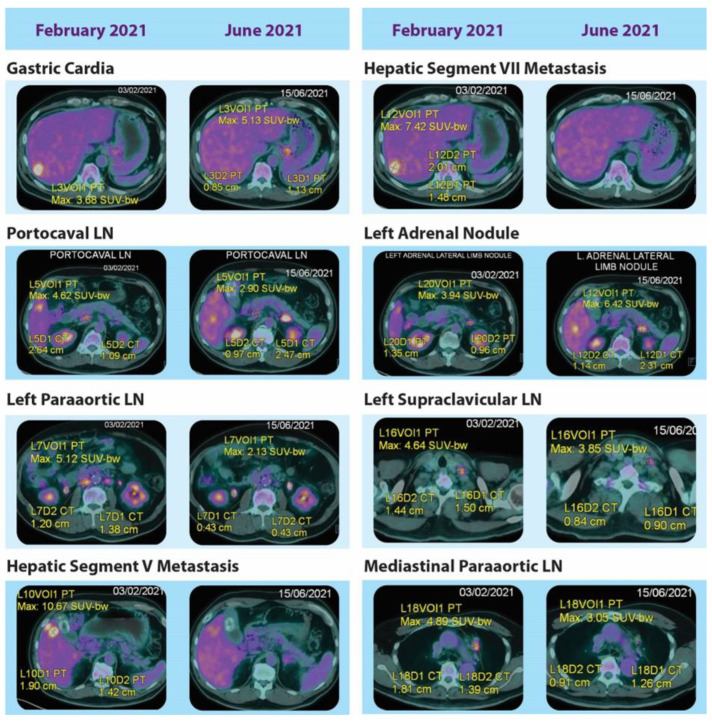
PET/CT images before and after 3 cycles of trastuzumab deruxtecan, showing varied responses in the primary and metastatic sites.

The 3L treatment with T-DXd achieved satisfactory tumor control, as displayed in the PET/CT findings at 2 months (Figure 1). Symptoms were stable and toxicities were minimal. The patient found it more manageable to visit the hospital once every 3 weeks for an injection compared with the previous regimens, especially during the COVID-19 pandemic. Because T-DXd was a novel treatment in GC, the patient was monitored closely, which included chest X-rays and blood tests (liver enzymes, bilirubin, blood counts, tumor markers, etc.) at every cycle for the first three cycles. No significant cardiovascular or pulmonary toxicities were observed.

**Figure 2:**
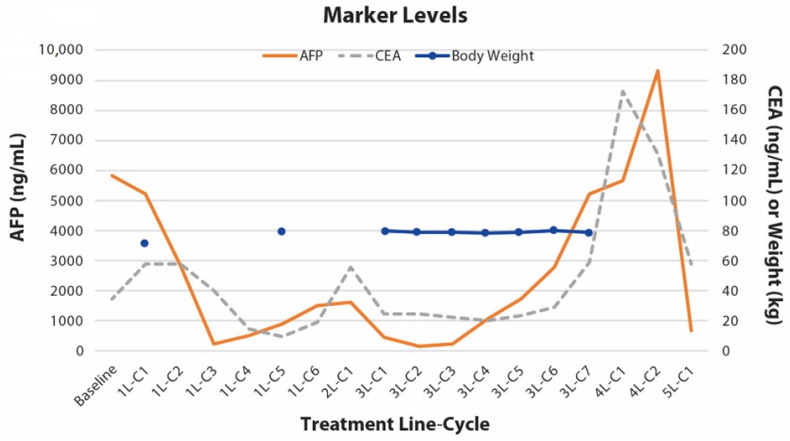
Tumor marker levels during treatment.

At around the fourth T-DXd cycle, tumor markers rebounded (Figure 2). Radiographic progression was confirmed after the seventh cycle (Supplementary Figure S2), and the patient was switched to the immune checkpoint inhibitor (ICI) nivolumab plus TAS-102 (trifluridine/tipiracil). Two months later, due to further progression, the tumors were “re-challenged” with paclitaxel and ramucirumab, in combination with trastuzumab, as a fifth-line treatment. AFP and CEA levels were markedly reduced after one cycle (Figure 2). Unfortunately, the patient’s condition continued to deteriorate, and he passed away in late December 2021, 16 months after the initial diagnosis.

## 3. Discussion

Given the patient’s symptomatic advanced-stage cancer diagnosed de novo, his marginal performance status, and the poor prognosis, the objectives of the systemic palliative treatment were focused on effectively deterring disease progression, and ameliorating his symptoms to improve performance status and quality of life throughout the anticipated multiple lines of therapy.

For HER2+ unresectable mGC, the standard 1L chemotherapy is a platinum-fluoropyrimidine doublet with the addition of trastuzumab [1,14]. For our patient, S-1 was selected over 5-FU or capecitabine, as the former has been associated with lower grade 1–2 toxicities and grade 3–4 dehydration when compared with 5-FU-based regimens (in both Western and Asian patients), and lower rates of grade 1–2 hand–foot syndrome and grade 3–4 neutropenia when compared with capecitabine-based regimens (in Asian patients) [15]. The platinum agent oxaliplatin was preferred over cisplatin because of lower nephrotoxicity and neurotoxicity, in accordance with the U.S. National Comprehensive Cancer Network gastric cancer guidelines [1]. In recent years, the efficacy and safety of the SOX + trastuzumab combination have been validated in Asian HER2+ GC patients [16,17].

Not long after starting 2L treatment, the patient experienced severe peripheral neuropathy (Table 2), which is a known adverse effect of both oxaliplatin and paclitaxel [18]. At 3L, the patient received T-DXd for 5 months (seven cycles) before progression, which was similar to the median treatment duration (4.6 months) reported in the DESTINY-Gastric01 T-DXd arm [5]. Our patient had a large total tumor burden of ≥10 cm in measurable diameters of all lesions, both at presentation and before 3L treatment, which was observed in about 1 in 5 patients in the DESTINY-Gastric01 study [5]. Given the aggressive GAED subtype and extensive metastatic burden, the patient received T-DXd for 4–5 months without progression, and lived for 9 months since 3L therapy initiation, which may be somewhat less than the median PFS and OS reported in DESTINY-Gastric01 (5.6 and 12.5 months, respectively) [5].

During 3L T-DXd, non-hematologic toxicities were minimal. Hematologic toxicities consisted of grade 3 neutropenia and grade 3 anemia, which were the two most common grade 3 or 4 adverse events in DESTINY-Gastric01 (T-DXd arm vs. chemotherapy arm: 51% vs. 24% for grade ≥ 3 neutropenia, and 38% vs. 23% for grade ≥ 3 anemia) [5]. No thrombocytopenia was observed in our patient. The patient was given a granulocyte-colony-stimulating factor for neutropenia management, but no blood transfusion was needed for the anemia. After failing 3L and 4L therapies, the patient could still retain an adequate level of physical performance and organ functions to continue further therapy (instead of choosing best supportive care).

The patient lived alone, and was concerned about whether he could continue his daily activities on his own. During 1L and 2L therapies, he found it difficult to adhere to frequent hospital visits, particularly during the COVID-19 pandemic. With relatively few toxicities and a 3-weekly dosing schedule, the T-DXd regimen helped to maintain his normal daily activities and a good quality of life. During most the 3L treatment period, the patient was able to make short everyday commutes independently.

Since the patient was positive for both HER2 and PD-L1 expression, an important consideration was the selection and sequencing of targeted treatments. For HER2+ GC, the 1L use of trastuzumab in combination with chemotherapy was well-established by the phase 3 TOGA trial [19]. At the time of treatment initiation in August 2020, evidence for using ICIs in 1L therapy for mGC was limited to HER2-negative patients only, supported by the CheckMate 649 study of nivolumab [20]. Our patient was therefore treated with 1L trastuzumab in combination with S-1 and oxaliplatin, without the addition of an ICI. For subsequent treatment, results from the DESTINY-Gastric01 study of 3L T-DXd (published in June 2020) showed a high disease-control rate of 86% in the T-DXd group (including 9% of patients reaching complete response and 42% with partial response), compared with 62% in the control group (0% with a complete response, and 14% with a partial response) [5]. Based on this evidence, the patient was switched to T-DXd upon failure of 2L paclitaxel plus ramucirumab.

The most recent evidence from the KEYNOTE-811 and DESTINY-Gastric02 trials offers a different treatment sequence from the above. On the one hand, results of KEYNOTE-811, which were presented in June and published in December 2021 [21,22], demonstrated significant ORR benefits of upfront pembrolizumab in combination with trastuzumab and chemotherapy for HER2+ mGC, compared with trastuzumab and chemotherapy alone, including an objective response of 74.4% vs. 51.9% in the two groups, respectively [21,22]. Based on ORR data from KEYNOTE-811, the U.S. FDA approved the addition of pembrolizumab to the combination of chemotherapy and trastuzumab in the 1L treatment of HER2+ mGC [23]. On the other hand, while the FDA approval for T-DXd was based on the 3L-setting trial DESTINY-Gastric01 [5,6], a single-arm, phase 2 study (DESTINY-Gastric02) explored the benefits of 2L T-DXd in HER2+ mGC patients who have failed a prior trastuzumab-based therapy [24,25]. Results were presented at the European Society for Medical Oncology Congress in September 2022 [25]. Among 79 HER2+ GC patients treated with 2L T-DXd, the confirmed ORR was previously reported to be 38% [24], and the newly reported median OS and median PFS were 12.1 months and 5.6 months, respectively [25]. European Medicines Agency approval for the study was given soon after [26]. Taking these results together, future cases of mGC expressing both HER2 and PD-L1 may benefit from upfront pembrolizumab in combination with trastuzumab and chemotherapy, followed by 2L T-DXd.

## 4. Conclusions

A patient with de novo stage 4 HER2+ GAED received 3L T-DXd for seven cycles. Tumor control was satisfactory, toxicities were minimal, and quality of life was well preserved. The patient retained acceptable physical performance to receive subsequent lines of therapies, and he still showed a tumor marker response to 5L trastuzumab-based chemotherapy. Further research and discourse are warranted to establish a consensus on the optimal position of T-Dxd in the sequencing of treatments for patients with HER2+ mGC, particlulary GAED, who progressed on trastuzumab.

## Figures and Tables

**Table 1:** Lines of treatment and responses.

Line	Treatment	Start Date	Cycles Completed	Best Response
1st	SOX + trastuzumab	September 2020	6	Partial response ^1,2^
2nd	Paclitaxel + ramucirumab	February 2021	1	Tumor marker response ^3^
3rd	Trastuzumab deruxtecan	April 2021	7	Stable disease ^1,2^
4th	Nivolumab + TAS-102	September 2021	Nivolumab: 5 TAS-102: 2	Progressive disease
5th	Paclitaxel + ramucirumab + trastuzumab	December 2021	1	Tumor marker response ^3^

^1^ For radiological images, see Figure 1 and Supplementary Figures S1 and S2. ^2^ <6 months. ^3^ Not radiographically assessed because of the short treatment duration.

**Table 2:** Observed toxicities and their management. CTCAE = common terminology criteria for adverse events; G-CSF = granulocyte-colony stimulating factor; NR = not required.

Line	Treatment Agents	Treatment Duration (Months)	Treatment-Related Toxicities	CTCAE Grade	Management
1st	S-1 + oxaliplatin + trastuzumab	5	Fatigue	1	NR
			Anemia	1	
			Peripheral neuropathy	2	
2nd	Paclitaxel + ramucirumab	1	Peripheral neuropathy	3	Discontinued regimen
3rd	Trastuzumab deruxtecan	5	Anemia	3	NR
			Neutropenia	3	G-CSF, with a total of 2 weeks in dose delays
			Thrombocytopenia	0	NR
4th	Nivolumab + TAS-102	3	Anemia	3	NR
			Neutropenia	2	G-CSF
			Thrombocytopenia	1	NR
5th	Paclitaxel + ramucirumab	1	Anemia	1	NR

## Data Availability

Further details may be available upon request to the corresponding author, subject to legal and privacy restrictions.

## Supplementary Materials

The following supporting information can be downloaded at https://www.mdpi.com/article/10.3390/life13091851/s1. Figure S1: PET/CT images before and after 6 cycles of upfront SOX plus trastuzumab, demonstrating apparent tumor size reductions; Figure S2: PET/CT images after 3 cycles of trastuzumab deruxtecan and before switching to fourth-line treatment, showing disease progression.

## References

[1] Gastric Cancer, Version 2.2022, 11 January 2022—NCCN Clinical Practice Guidelines in Oncology (NCCN Guidelines®). https://www.nccn.org/professionals/physician_gls/pdf/gastric.pdf.

[2] Wilke H., Muro K., Van Cutsem E., Oh S.C., Bodoky G., Shimada Y., Hironaka S., Sugimoto N., Lipatov O., Kim T.Y. (2014). Ramucirumab plus paclitaxel versus placebo plus paclitaxel in patients with previously treated advanced gastric or gastro-oesophageal junction adenocarcinoma (RAINBOW): A double-blind, randomised phase 3 trial. Lancet Oncol..

[3] Hecht J.R., Bang Y.J., Qin S.K., Chung H.C., Xu J.M., Park J.O., Jeziorski K., Shparyk Y., Hoff P.M., Sobrero A. (2016). Lapatinib in combination with capecitabine plus oxaliplatin in human epidermal growth factor receptor 2-positive advanced or metastatic gastric, esophageal, or gastroesophageal adenocarcinoma: TRIO-013/LOGiC—A randomized phase III trial. J. Clin. Oncol..

[4] Thuss-Patience P.C., Shah M.A., Ohtsu A., Van Cutsem E., Ajani J.A., Castro H., Mansoor W., Chung H.C., Bodoky G., Shitara K. (2017). Trastuzumab emtansine versus taxane use for previously treated HER2-positive locally advanced or metastatic gastric or gastro-oesophageal junction adenocarcinoma (GATSBY): An international randomised, open-label, adaptive, phase 2/3 study. Lancet Oncol..

[5] Shitara K., Bang Y.J., Iwasa S., Sugimoto N., Ryu M.H., Sakai D., Chung H.C., Kawakami H., Yabusaki H., Lee J. (2020). Trastuzumab deruxtecan in previously treated HER2-positive gastric cancer. N. Engl. J. Med..

[6] FDA Approves Fam-Trastuzumab Deruxtecan-nxki for HER2-Positive Gastric Adenocarcinomas. https://www.fda.gov/drugs/resources-information-approved-drugs/fda-approves-fam-trastuzumab-deruxtecan-nxki-her2-positive-gastric-adenocarcinomas.

[7] Aoki M., Iwasa S., Boku N. (2021). Trastuzumab deruxtecan for the treatment of HER2-positive advanced gastric cancer: A clinical perspective. Gastric Cancer.

[8] Alrhmoun S., Sennikov S. (2022). The role of tumor-associated antigen HER2/neu in tumor development and the different approaches for using it in treatment: Many choices and future directions. Cancers.

[9] Murakami T., Yao T., Mitomi H., Morimoto T., Ueyama H., Matsumoto K., Saito T., Osada T., Nagahara A., Watanabe S. (2016). Clinicopathologic and immunohistochemical characteristics of gastric adenocarcinoma with enteroblastic differentiation: A study of 29 cases. Gastric Cancer.

[10] Akazawa Y., Saito T., Hayashi T., Yanai Y., Tsuyama S., Akaike K., Suehara Y., Takahashi F., Takamochi K., Ueyama H. (2018). Next-generation sequencing analysis for gastric adenocarcinoma with enteroblastic differentiation: Emphasis on the relationship with hepatoid adenocarcinoma. Hum. Pathol..

[11] Yatagai N., Saito T., Akazawa Y., Hayashi T., Yanai Y., Tsuyama S., Ueyama H., Murakami T., Watanabe S., Nagahara A. (2019). TP53 inactivation and expression of methylation-associated proteins in gastric adenocarcinoma with enteroblastic differentiation. Virchows Arch..

[12] Yamazawa S., Ushiku T., Shinozaki-Ushiku A., Hayashi A., Iwasaki A., Abe H., Tagashira A., Yamashita H., Seto Y., Aburatani H. (2017). Gastric cancer with primitive enterocyte phenotype: An aggressive subgroup of intestinal-type adenocarcinoma. Am. J. Surg. Pathol..

[13] Kwon M.J., Byeon S., Kang S.Y., Kim K.M. (2019). Gastric adenocarcinoma with enteroblastic differentiation should be differentiated from hepatoid adenocarcinoma: A study with emphasis on clear cells and clinicopathologic spectrum. Pathol. Res. Pract..

[14] Lordick F., Carneiro F., Cascinu S., Fleitas T., Haustermans K., Piessen G., Vogel A., Smyth E.C., Committee E.G. (2022). Gastric cancer: ESMO clinical practice guideline for diagnosis, treatment and follow-up. Ann. Oncol..

[15] Ter Veer E., Ngai L.L., Valkenhoef G.V., Mohammad N.H., Anderegg M.C.J., van Oijen M.G.H., van Laarhoven H.W.M. (2017). Capecitabine, 5-fluorouracil and S-1 based regimens for previously untreated advanced oesophagogastric cancer: A network meta-analysis. Sci. Rep..

[16] Takahari D., Chin K., Ishizuka N., Takashima A., Minashi K., Kadowaki S., Nishina T., Nakajima T.E., Amagai K., Machida N. (2019). Multicenter phase II study of trastuzumab with S-1 plus oxaliplatin for chemotherapy-naive, HER2-positive advanced gastric cancer. Gastric Cancer.

[17] Xu D., Zhang Z., Zhang S., Fang X., Wang L., Li Q. (2021). Efficacy of trastuzumab combined with SOX or IP chemotherapy regimen in the treatment of advanced gastric cancer. J. BUON.

[18] Pachman D.R., Qin R., Seisler D., Smith E.M., Kaggal S., Novotny P., Ruddy K.J., Lafky J.M., Ta L.E., Beutler A.S. (2016). Comparison of oxaliplatin and paclitaxel-induced neuropathy (Alliance A151505). Support. Care Cancer.

[19] Bang Y.J., Van Cutsem E., Feyereislova A., Chung H.C., Shen L., Sawaki A., Lordick F., Ohtsu A., Omuro Y., Satoh T. (2010). Trastuzumab in combination with chemotherapy versus chemotherapy alone for treatment of HER2-positive advanced gastric or gastro-oesophageal junction cancer (ToGA): A phase 3, open-label, randomised controlled trial. Lancet.

[20] Janjigian Y.Y., Shitara K., Moehler M., Garrido M., Salman P., Shen L., Wyrwicz L., Yamaguchi K., Skoczylas T., Campos Bragagnoli A. (2021). First-line nivolumab plus chemotherapy versus chemotherapy alone for advanced gastric, gastro-oesophageal junction, and oesophageal adenocarcinoma (CheckMate 649): A randomised, open-label, phase 3 trial. Lancet.

[21] Janjigian Y.Y., Kawazoe A., Yanez P., Li N., Lonardi S., Kolesnik O., Barajas O., Bai Y., Shen L., Tang Y. (2021). The KEYNOTE-811 trial of dual PD-1 and HER2 blockade in HER2-positive gastric cancer. Nature.

[22] Janjigian Y.Y., Kawazoe A., Yanez P.E., Luo S., Lonardi S., Kolesnik O., Barajas O., Bai Y., Shen L., Tang Y. (2021). Pembrolizumab plus trastuzumab and chemotherapy for HER2+ metastatic gastric or gastroesophageal junction (G/GEJ) cancer: Initial findings of the global phase 3 KEYNOTE-811 study. J. Clin. Oncol..

[23] FDA Grants Accelerated Approval to Pembrolizumab for HER2-Positive Gastric Cancer. https://www.fda.gov/drugs/resources-information-approved-drugs/fda-grants-accelerated-approval-pembrolizumab-her2-positive-gastric-cancer.

[24] Van Cutsem E., Di Bartolomeo M., Smyth E., Chau I., Park H., Siena S., Lonardi S., Wainberg Z.A., Ajani J.A., Chao J. (2021). LBA55 primary analysis of a phase II single-arm trial of trastuzumab deruxtecan (T-DXd) in Western patients (Pts) with HER2-positive (HER2+) unresectable or metastatic gastric or gastroesophageal junction (GEJ) cancer who progressed on or after a trastuzumab-containing regimen. Ann. Oncol..

[25] Ku G.Y., Di Bartolomeo M., Smyth E., Chau I., Park H., Siena S., Lonardi S., Wainberg Z.A., Ajani J.A., Chao J. (2022). 1205MO updated analysis of DESTINY-Gastric02: A phase II single-arm trial of trastuzumab deruxtecan (T-DXd) in Western patients (Pts) with HER2-positive (HER2+) unresectable/metastatic gastric/gastroesophageal junction (GEJ) cancer who progressed on or after trastuzumab-containing regimen. Ann. Oncol..

[26] Enhertu|European Medicines Agency. https://www.ema.europa.eu/en/medicines/human/EPAR/enhertu#authorisation-details-section.

