# Supplementary figures and images for: Outcomes of Third-Line Trastuzumab Deruxtecan in a Patient with De Novo Stage 4 HER2-Positive Gastric Adenocarcinoma with Enteroblastic Differentiation: A Case Report
Source: Life (Basel). 2023 Aug 31;13(9):1851. doi: 10.3390/life13091851 (PMC10533056; doi:10.3390/life13091851)

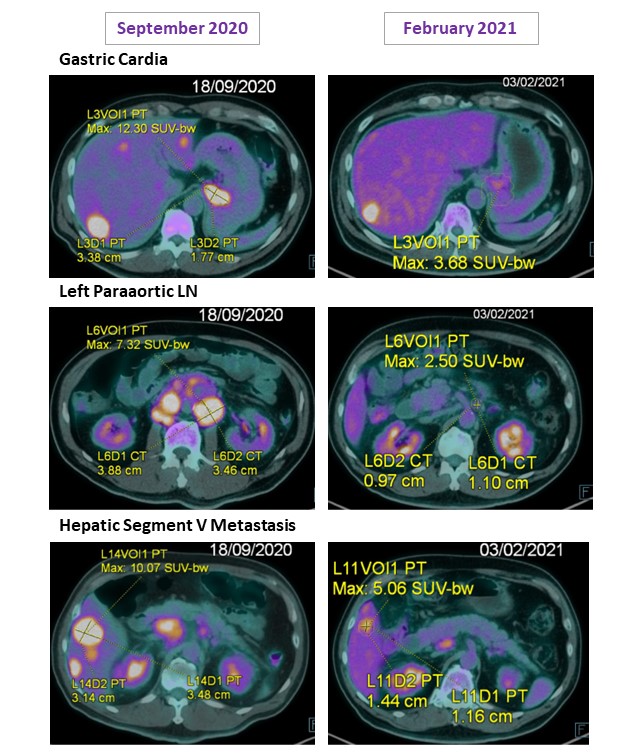

Supplement: Supplementary file 1 [file life-13-01851-s001.zip › Figure S1.jpg]

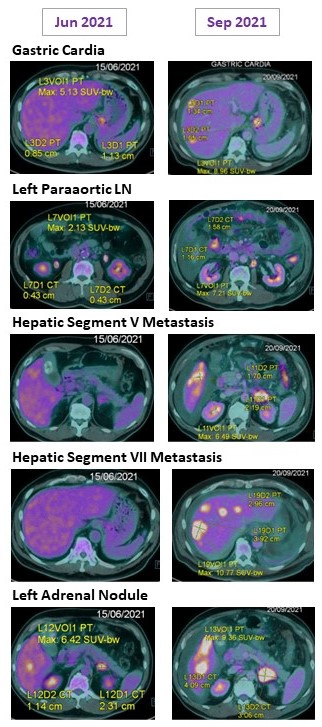

Supplement: Supplementary file 1 [file life-13-01851-s001.zip › Figure S2.jpg]
